# Does an integrated school eye health delivery model perform better than a vertical model in a real-world setting? A non-randomised interventional comparative implementation study in Zanzibar

**DOI:** 10.1136/bjo-2022-321752

**Published:** 2022-11-14

**Authors:** Ving Fai Chan, Elodie Yard, Eden Mashayo, Damaris Mulewa, Lesley Drake, Carlos Price-Sanchez, Fatma Omar

**Affiliations:** 1 Centre for Public Health, Queen's University Belfast, Belfast, UK; 2 Brien Holden Vision Institute Foundation (Africa) Trust, Durban, South Africa; 3 Partnership for Child Development, Imperial College London, London, UK; 4 Ministry of Health, Zanzibar, United Republic of Tanzania

**Keywords:** Child health (paediatrics), Epidemiology

## Abstract

**Background:**

Few studies on school eye health programmes have shown they were cost-effective. We compared the performance (Reach, Effectiveness, Adoption, Implementation and Maintenance (RE-AIM)) between an integrated model (IM) and a vertical model (VM) of school eye health delivery in Zanzibar.

**Methods:**

The set of RE-AIM performance indicators of the IM (n=9) and VM (n=10) cohorts was compared. The VM implemented only the eye health interventions, while the IM had the eye health interventions conducted within the school feeding programme. Semistructured interviews were conducted with 36 stakeholders to understand the challenges and outcomes experienced when implementing both models.

**Results:**

The IM achieved higher screening coverage, voluntary follow-up rate, screening validity and spectacle compliance than VM. This was due to effective coordination between implementers, motivated teachers to prevent vision problems and related negative impacts in children, and activities implemented timeously post-training. Both models recorded low wearing compliance. All schools in the IM cohort completed screening activities, but two schools in the VM cohort did not. Both models ceased activities after the funding stopped. Local stakeholders emphasised that evidence from this study can be used to advocate for more resources for children’s eye health.

**Conclusions:**

The IM cohort achieved better reach, effectiveness, adoption rate and implementation performance than the VM cohort. The poor maintenance performance indicators in both arms postfunding call for improvement to the implementation strategy to ensure the sustainability of school eye health. In the optics of scaling up, an integrated approach is recommended.

**WHAT IS ALREADY KNOWN ON THIS TOPIC**

- Findings from Zanzibar showed that delivering school eye health using an integrated model is more cost-effective than the vertical model. Few studies explored the readiness to implement integrated school eye health programmes in low-resource settings.

**WHAT THIS STUDY ADDS**

- We demonstrated that the integrated school eye health delivery model achieved better reach, effectiveness, adoption rate and implementation performance than the vertical delivery model.

**HOW THIS STUDY MIGHT AFFECT RESEARCH, PRACTICE OR POLICY**

- Our findings demonstrated the potential to deliver large-scale public health benefits in a real-world setting using limited resources through an integrated school eye health delivery model.

## Introduction

Vision impairment is a major burden to children, carers and societies.^1^ It is estimated that 19 million children have poor vision, and 1.4 million are blind.^1^ Vision impairment in children can contribute to low self-esteem,^2^ poor cognitive performance,^3^ distress,^4^ anxiety and depression,^5^ and restricted future economic productivity.^6 7^ The most common eye conditions among Zanzibari children, such as uncorrected refractive error and eye infections, are preventable and treatable. In Zanzibar, about 22 000 children (5% of 6–12 years old) need conjunctivitis treatment or spectacles (Ministry of Health (MoH) monitoring data, unpublished). The reasons for unmet needs include a lack of awareness about the eye problems children face, a lack of trust in available treatments, as well as many cultural and social barriers to remediation among children and parents.^8^

Recognising the considerable socioeconomic consequences of untreated eye conditions, the MoH in Zanzibar established a Primary Healthcare Programme to reduce the societal burdens of preventable eye diseases.^9^ The MoH now supports free basic eye examinations, the distribution of eye-drops and refractive services provided at all levels of healthcare.^9^ The MoH also aims to make eye health screening in children an essential part of the school health programme because screenings are simple to conduct and are not resource-intensive.^10^ However, these school eye health programmes are historically ad hoc and funded on a short-term basis by non-governmental organisations. Consequently, they tend to end abruptly when funding ceases, limiting their sustainability and long-term impact. The current school health programme focuses on food and nutrition. Given the importance of ocular nutrition to child development, the Government of Zanzibar aims to integrate school eye health programmes into pre-existing school nutrition programme (SFP) to address long-standing child eye health needs.

Economic modelling in Africa, Europe and Asia,^11^ and cost-effectiveness analyses of school vision screening in Asia,^12 13^ suggest that screening for and correcting refractive error in children within school settings is cost-effective and improves health outcomes. However, limited evidence exists on the optimal delivery models. Hence in 2017, two organisations—one with a focus on education, health and nutrition in school-aged children, and an international eye care non-governmental organisation—collaborated to pilot an integrated School Eye Health Programme in the two islands of Zanzibar to address this knowledge gap. In the integrated model (IM), eye health is integrated into mainstream school health programmes where a nutrition programme already exists. In the vertical model (VM), eye health is offered through teachers as a stand-alone intervention. The goal of this pilot was to assess the efficacy, in terms of performance and cost-effectiveness, of the two models and subsequently identify the potential pathway to integration.

More specifically, the pilot consisted of three studies. First, we aimed to assess the cost-effectiveness of the two models.^14^ In this study, we showed that in 6 months, VM used 1.2 times more resources per child screened than the IM, and the cost per child identified in the VM is twice that of the IM; thus, the IM is highly cost-effective. Second, we aimed to assess performance and outcomes on eye health indicators to eventually inform decision-making by the Government of Zanzibar (which is the focus of this article). Lastly, we conducted semistructured interviews with stakeholders to determine pathways towards fully integrating school eye health into the mainstream school health programme.^15^

## Methods

### Study design

A non-randomised intervention comparative study was conducted to determine the performances of an integrated (IM) and vertical (VM) school eye health programme. The school eye screening programme was implemented in 19 schools from April 2017 to October 2017 in Unguja (North A and South District) and Pemba Island (Micheweni and Mkoani District). Before the study began, permission to conduct the screening and research was obtained from the Ministry of Education and Vocational Training and school principals. Parental consent and children’s assent were obtained before the children participated in the screening. Informed consent was obtained from key informants before participating in the semistructured interviews. We reported our findings according to the Standards for Reporting Implementation Studies statements. It was impossible to blind the school authorities and investigators to the intervention (IM or VM); they were blinded to the study’s hypothesis.

### Intervention and intervention strategy

The intervention of this programme has been described previously.^14 15^ Each principal nominated two to three teachers (1 teacher to 300 students) for a 2-day training. In the VM cohort (10 schools), each teacher was trained on screening children’s distance vision at 6 m using a modified Snellen’s chart with a 6/12 cut-off and identifying obvious eye diseases (red eye, squint, eyes with discharge and cataract) with a torch. If the child could not identify at least 4 out of 5 letters on the 6/12 line, or/and they had obvious eye disease, the teachers would refer the child to the nearest eye clinics for further examination. Furthermore, the teachers were given a set of eye health education materials, which included posters to be displayed in the schools and brochures and eye health booklets to be used to teach about eye health during school health club sessions. The materials included six messages: (1) Eye and facial cleanliness are important to prevent red itchy eyes; (2) Nutritious food is important for healthy eyes; (3) Ways to prevent eye injuries; (4) What a squint is and the need for immediate referral; (5) What a cataract is and the need for immediate referral and (6) Information about reduced vision and the need for immediate referral. After the training, each teacher was provided with a kit that included a modified 5-letter Snellen’s chart with a 6/12 cut-off, a measuring tape, a screening record register, referral cards and health education material.

On top of the eye health training, teachers in the IM (nine schools) were also provided with a height and weight measuring machine and health education material on (1) what constitutes a balanced diet; (2) the importance of sanitation and hygiene with a focus on how to keep a school and home toilet clean and (3) signs of worm infestation in a child and the importance of deworming. They were trained to measure children’s height and weight, identify children with nutrition issues and those with body mass index, BMI <18 kg/m^2^ and BMI >30 kg/m^2^) and refer them to the nearest health centre for management.

In April 2017, 60 teachers (n=30 in both IM and VM) were trained to deliver the assigned interventions. Teachers conducted eye health screening, recorded all students screened and identified the students who required follow-up. The performance observation period was from April to January 2018. Children who failed the eye health screening were referred to a designated vision centre for vision management. The optometrist examined the referred children and managed their eye condition. The treatment provided at the vision centre included spectacle provision and basic eye medication. Any cases that could not be managed at the vision centre were referred to Muhimbili Hospital in Dar-es-Salaam for further management.

### Sampling procedure

The sampling framework employed for the study was based on the number and demography of the children enrolled in nine schools with an SFP in Zanzibar. We estimated that about 6000 children from the school register were enrolled in the nine selected schools (IM). To match this, 10 schools were selected for the VM, given their lower enrolment. Schools in both models were rural and had similar student attendance and distance to the nearest eye centres (within a service radius of 5 km). At the end of the intervention period, there were 11 978 children enrolled in the schools, with 6257 children in the IM and 5721 children in the VM (table 1).

**Table 1:** Characteristics of schools, children and teachers in the integrated and vertical models

School characteristics	Integrated model, n (%)	Vertical model, n (%)
	Total schools=10	Total schools=9
No of schools with distance to eye facilities <2 km	4 (40%)	4 (44.4%)
No of schools with distance to eye facilities 2–5 km	6 (60%)	5 (55.6%)
Location		
Urban	0 (0%)	0 (0%)
Rural	10 (100%)	9 (100%)
Children characteristics	Total children enrolled=6257	Total children enrolled=5721
Sex		
Boys	3127 (50.0%)	2960 (51.7%)
Girls	3130 (50.0%)	2761 (48.3%)
Previously had eye health screened or examined	18 (0.26%)	24 (0.42%)
History of parent/s worn spectacles or current spectacle wearers	26 (0.42%)	14 (0.25%)
Teacher characteristics	Total teachers=168	Total teachers=175
No of teachers working at the schools		
Male	62 (36.9%)	53 (30.3%)
Female	106 (63.1%)	122 (69.7%)
No of teachers trained in the programme	30 (17.9%)	30 (17.1%)
Previously trained in eye health	3 (10%)	4 (13.3%)
No previous experience in eye health	27 (90%)	26 (86.7%)

### Indicators and data collection

The Reach, Effectiveness, Adoption, Implementation and Maintenance (RE-AIM) framework^16^ guided this study. Informed by the indicators proposed by the School Health Integrated Programming guidelines,^17^ and the International Agency for the Prevention of Blindness Standard School Eye Health Guidelines for low-income and middle-income countries,^18^ the provisional indicators for each RE-AIM element were developed. To ensure shared decision making based on equal relationships and representation, interviews were held with stakeholders to design the study and agree on the final performance indicators (table 2).

**Table 2:** The indicators for the Reach, Effectiveness, Adoption, Implementation and Maintenance elements for the study

Element	Quantitative outcome	Qualitative outcome
Reach	The proportion of children screened	Reasons a high screening coverage achieve/not achieve
Effectiveness	The proportion of children who went for follow-up examination/postreferral The proportion of children wearing spectacles during an unannounced visit The proportion of children wearing spectacles full time	Enabling or inhibiting factors achieving the expected outcomes
Adoption	The proportion of schools that started eye health and health screening within 2 months after the programme was implemented The proportion of schools that completed screenings at the end of 6 months	Factors inhibit the complete adoption of the programme
Implementation	The proportion of children who fail eye health screening, when re-examined by the optometrist, have vision worse than 6/12 or obvious eye diseases (validity) The proportion of children who pass screening, when re-examined by the optometrist, have 6/12 vision or better and no obvious eye diseases (validity)	Factors influencing the implementation or lack of implementation of the programme
Maintenance	The proportion of schools that continued with the programme after funding had ended	Enabling and inhibiting factors for the continuation of the programme

All teachers who underwent training took a pretraining and post-training assessment. A list of children identified with eye conditions was compiled from the student eye screening register. The research coordinator visited the schools to collect the list monthly. The optometrist compiled a list of names of the children examined, and their diagnoses were recorded. Two months after the programme was implemented, a team of researchers conducted a monitoring trip to determine the number of schools that completed the activities, why any teachers had not completed screening and the eye health screening validity. Teachers who had not yet started the screening activities were requested to start, while those who had not completed screening activities were asked to continue. Six months after the programme was implemented, the local researchers returned to the schools to determine (1) the number of schools that completed screenings, (2) the number and reasons of children not going for follow-up eye examinations at the eye clinics, (3) the spectacle-wearing compliance and (4) the reasons for not wearing their spectacles.

A series of semistructured interviews were conducted with a purposively selected sample of implementors from the MoH (n=4) and Ministry of Education and Vocational Training (n=8) and teachers (n=24) to capture and understand individual and organisational challenges and outcomes experienced during programme implementation. In January 2018, headteachers were contacted to determine if the schools continued with the programme after funding had ended and the reasons for continuing or discontinuing.

## Results

### Reach

**Figure 1:**
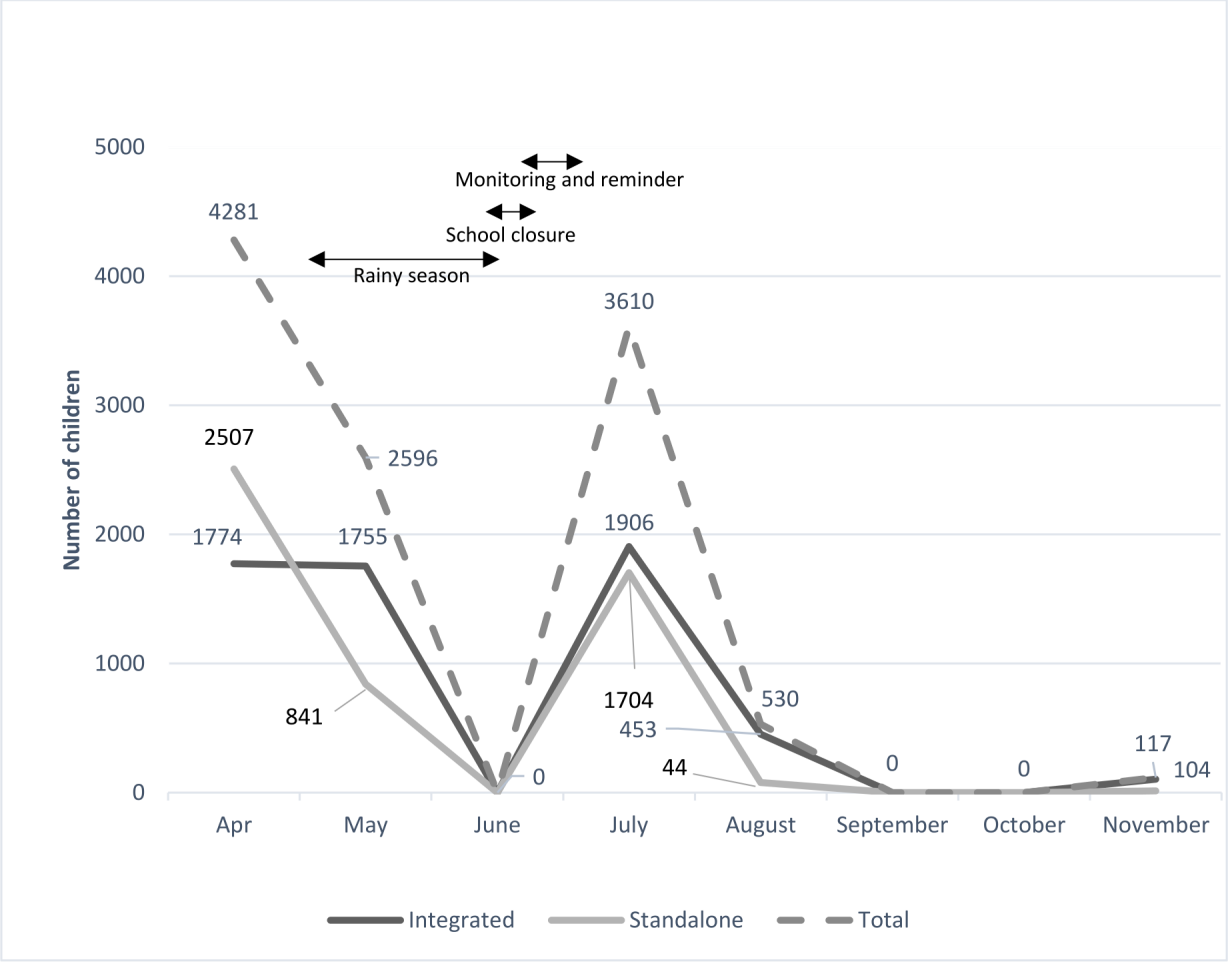
Number of children screened from April to November 2017.

Both models achieved a high screening coverage (≥ 90%) with IM achieving a slightly higher coverage (n=5992/6257, 96%; 95% CI 92.1% to 100%) than VM (n=5142/5721=90%; 95% CI 85.7% to 95.8%) (previously reported^14^ for cost-effective analysis). The high coverage resulted from a high rate of school attendance, effective planning, coordination and implementation of the interventions between programme partners, schools and teachers. Figure 1 shows that both models observed a similar trend throughout the study months: screening rates were highest in April and decreased steadily to no children screened in June, then peaked again in July and decreased to zero from September onwards.

Over 90% of the total number of children were screened within 4 months of initiating the programme, peaking in April and July. In June, no screening was conducted as all schools were closed because of monsoon flooding. Respondents highlighted that programme implementers must strategically schedule school health activities effectively and efficiently to ensure minimal interruptions. [*‘The interruption of the eye health screening, however, should serve as a reminder there are also unpredictable natural disasters that may affect programme implementation, and mitigation plan must be in place when such situations arise.*’ (Ministry of Health Representative 20)]

### Effectiveness

The IM recorded a higher proportion of children (n=77/121, 63.6%; 95% CI 57.5% to 69.7%) seeking follow-up eye examinations at the nearest vision centre than the VM (n=46/100, 46%; 95% CI 35% to 57%). The main reasons for not going for eye examination among children in both models were lack of financial means and not being aware of the need for a further eye examination (table 2). The teachers from six schools in the IM took the initiative to organise trips to the vision centres so that the optometrist could examine the children. In return, the parents paid for the transport fees.

Of the 61 children who received spectacles, more children in the IM (n=22/31, 71%) than the VM (n=4/30, 13.3%) wore their spectacles during the spectacle-compliance monitoring visit. Only one child was wearing them full time in the IM, and none wore them all the time in the VM. The main reasons for the part-time wear of spectacles in both models were that their friends teased them (IM, n=12, 33.3%; VM n=2, 66.7%). Another reason for low compliance in the IM was that the wearers did not think they needed to wear the spectacles all the time (n=11, 30.6%) (table 2). Another 37 children (n=29, 19% in IM and n=8, 5.3% in the VM) were treated with eye medication due to other eye morbidities (table 3).

**Table 3:** Children’s reasons for not going for an eye examination at the eye clinics, their daily spectacle-wearing time and reasons for not wearing their spectacles more frequently

	Integrated model	Vertical model
	No of children (%)	No of children (%)
Reasons for not going for an eye examination at the eye clinics		
Financial constraint	8 (42.1)	15 (26.8)
Not asked to go to the hospital	8 (42.1)	19 (33.9)
Perceived no eye problem	0 (0)	3 (5.4)
Hospital too far	1 (5.3)	9 (16.1)
Just did not want to go	0 (0)	4 (7.1)
Parents too busy to bring them to the hospital	1 (5.3)	2 (3.6)
Parents ignored the referral letter	1 (5.3)	4 (7.1)
Daily spectacle-wearing time		
Only during school hours	20 (90.9)	3 (75)
Occasionally	1 (4.5)	0 (0)
Never	0 (0)	1 (25)
Whole day	1 (4.5)	0 (0)
Reasons for not wearing spectacles more frequently		
Friends teased me when I wore them	12 (33.3)	2 (66.7)
I feel uncomfortable wearing spectacles	7 (19.4)	1 (33.3)
I do not particularly appreciate wearing them	6 (12.7)	0 (0)
I do not have to wear them often	11 (30.6)	0 (0)

### Adoption

Two months after the training was completed when the monitoring was conducted, all schools in the IM had started the eye health screening activities, while three schools in the VM had not begun the interventions due to the need to complete the term’s syllabus. It was highlighted that there is a need for

‘… consultations to be conducted with headteachers and teachers to identify the most suitable time for screening activity, even though some teachers have indicated that weekends at the beginning of the school academic year is the best time’.

Headteacher 4, South District

At the end of the project, all schools in the IM completed the activities, while two schools in the VM did not as they felt that *‘it was too late to catch up*’ (Teacher 1, Micheweni; Teacher 1, North A). However, the main enabling factor to complete the activities was that the teachers *‘felt happy that they could contribute to the early identification of children with vision problems before it negatively affected the child’s learning experience*’ (Teacher 2, South District; Teacher 1, Mkoani)

### Implementation

Before the eye health screening training began, 48 of the 60 teachers scored more than 75% on the training assessment, while all scored more than 75% at the end of the training. Two months after programme implementation, we found that 76% and 58% of those who failed eye health screening in the IM and VM had either vision less than 6/12 or obvious eye disease when re-examined by the optometrist. The main reason for this average validity was that many teachers *‘forgot the procedures as they started the screening two to three weeks after the training’ (Teacher 4, Micheweni; Teacher 2, South District*). However, all the children who passed screening had vision better than 6/12 or no obvious eye disease when re-examined by the optometrist. Respondents also revealed an underlying suspicion of western medicine and public health initiatives that led parents to choose traditional healing methods over eye examinations at the hospital. Most Zanzibari *‘prefer medicinal plants provided by traditional healers over pills and tablets’ (Headteacher, North A; Ministry of Health Representative 2*) because *‘plants are more natural’ (District Education Officer 1*). Site observation also revealed the children’s rejection of such school initiatives when the eye health posters were removed or vandalised.

### Maintenance

In both models, no schools continued to conduct screening in the new academic year cycle of January 2018 after the funding ended in September 2017. The main reasons for not continuing with the screenings were that *‘no budget was allocated for the activities*’ (Teacher, Micheweni; Headteacher, North A) and that there was *‘no directive to resume activities*’ (District Education Officer). The teachers and headteachers stressed that to ensure continuation, *‘school eye health must be mainstreamed into the Ministry of Health agenda to ensure a budget is allocated for its integration into school health programme*’ (Headteacher 1, Mkoani) and that the findings from the current study can be used as *‘evidence to advocate for more resources for children’s eye health*’ (Ministry of Health representative 3)

A summary of study findings is shown in table 4.

**Table 4:** Performance of the integrated and vertical models and the enabling and inhibiting factors to their performances

Elements	Performance indicators	Integrated model	Vertical model	Remarks
Reach	The proportion of children screened per number of children enrolled at school	96%	90%	Enabling factor—good coordination Inhibiting factor—seasonal influences
Effectiveness	The proportion of children who went for follow-up examination/postreferral The proportion of children wearing spectacles during an unannounced visit The proportion of children wearing spectacles full time	63.6% 71% 4.5%	46% 13.3% 0%	Enabling factor—teachers organised transport to students for examination Inhibiting factor—lack of funds to procure spectacles; not aware of the need for examination Inhibiting factors—teased by friends and discomfort experienced with spectacles wear
Adoption	The proportion of schools that started eye health and health screening within 2 months after the programme was implemented The proportion of schools that completed screenings at the end of 6 months	100% 100%	70% 80%	Enabling factors—motivated teachers Inhibiting factors—screening schedule interrupt with teaching; too late to catch up after a delay
Implementation	The proportion of children who fail eye health screening, when re-examined by the optometrist, have vision worse than 6/12 or obvious eye diseases (Validity) The proportion of children who pass screening, when re-examined by the optometrist, have 6/12 vision or better and no obvious eye diseases (Validity)	76% 100%	58% 100%	Enabling factor: Immediate implementation post-training Inhibiting factor: Delayed implementation post-training
Maintenance	The proportion of schools that continued with the programme after funding had ended	0%	0%	Inhibiting factors: Unavailability of budget and directive to resume activities

## Discussion

This study aimed to compare the performance of integrated and vertical delivery of school eye health. We found that the IM achieved a higher coverage of eye health screening (IM=96%, VM=90%), voluntary follow-up rate (IM=63.6%, VM=46%) and spectacle-wearing rate (IM=71%, VM=13.3%) than VM. Furthermore, all the teachers in the IM completed their activities in 2 months and had higher validity (IM=76%, VM=58%) in eye health screening. These positive findings result from effective coordination between the implementing partners, motivated teachers willing to conduct eye health intervention and quick implementation of eye health screenings after the training in the IM.

Our study findings addressed the concern that an integrated approach might decrease performance due to diverted attention given to multiple health interventions.^19^ The implementation, coordination management and monitoring process can be complex and hamper the IM’s performance. However, the IM could promote optimum use of resources by using teachers’ time in targeting multiple conditions within one programme. Our current and previous^14^ findings support this assumption.

There are a few challenges that remain. First, the timing of programme implementation is of critical importance. We recommend that screening be completed by February to avoid disruption from collecting data in the rainy, harvesting and Ramadhan seasons. Furthermore, the screening validity in IM is higher than findings from Vietnam^20^ and Iran.^21^ Screening should begin shortly after teachers receive training to ensure they can promptly apply their training and improve screening validity.

It was observed that the number of children who voluntarily went for follow-up management was average in both models, though slightly higher in the IM. The financial barrier was the main issue—a common barrier to service uptake in most low-income and middle-income countries.^22^ With a Gross Domestic Product per capita of US$749, child eye health may be a lower priority in a household than basic living necessities in Zanzibar. Moreover, traditional medicine has been known to be embedded in the Zanzibari culture. Traditional medicine in Zanzibari is usually provided by traditional healers using medicinal plants.^23^ Hence, improving eye health awareness among parents, teachers and children is critical to ensure a greater understanding of the importance of seeking eye treatment at eye facilities to avoid vision impairment. Although health posters and brochures have successfully increased service uptake in other contexts,^24^ we observed that the posters were removed or vandalised. This indicates the need to develop a locally acceptable eye health education strategy in collaboration with local stakeholders.

The spectacle wear was low in both models compared with previous studies, which reported higher spectacle compliance among schoolchildren in Oman (71.6%),^25^ in India (29.5%)^26^ and in Mexico (13.4%).^27^ To avoid children teasing each other due to spectacle wear, teachers must be trained to identify potential cases of teasing and develop counselling skills, complementing a health sensitisation programme. Moreover, a change of spectacle-wearing habits will require constant health education among the children, teachers and parents to understand the importance of wearing them full time to avoid blinding conditions, such as lazy eyes.

None of the schools continued their activities after the funds ended. This shortcoming highlighted the need to advocate for integrating eye health into school health programmes, as recommended by the local stakeholders. This would result in greater sustainability. Furthermore, our previous study^13^ also showed that the cost per child screened for IM was only US$1.23. The cost per child identified for having eye morbidity was US$24.76, making IM a more cost-effective school eye health delivery screening than VM. This demonstrates great opportunities for cost savings and suggests that it makes economic sense to implement the IM in our context.

Limitations of the study should also be acknowledged. The fact that the school eye health programme was integrated into schools where a school health and nutrition programme exists might have introduced several confounders. For example, the children and teachers in the IM might be more knowledgeable about health issues and hence performed better than the VM. Second, our study employed an implementation science methodology on a pilot programme. A large-scale cluster randomised trial with a longer-term follow-up could be conducted to determine the performance and sustainability of the programme. Third, issues on maintaining teachers’ motivation and more effective education strategies will need to be studied further. Currently, a study on arts-based eye health education strategy to improve child eye health service uptake is underway in Zanzibar.^28^

### Conclusion

Despite the complexity of a programme that consists of more than one health intervention, the IM achieved better reach, effectiveness and adoption rate performance in eye health than the VM. These findings are mainly due to effective planning, coordination and implementation of the interventions between the implementers, motivated teachers and activities implemented timeously post-training. In both models, the eye health intervention ceased when the funding stopped. Recommendations for an eye health programme scale-up include strong coordination between the implementers through participatory planning, a tailored health sensitisation strategy and messaging, as well as a sustainability plan that is codeveloped with local stakeholders.

## Data Availability

Data are available in a public, open access repository. Data are available in a public, open access repository. The datasets generated and/or analysed during the current study are available in the Queen’s University Belfast PURE repository, (DOI:10.17034/04cc7ddf-ce69-49ae-9876-e5dfdcedb545).
